# DrugMechDB: A Curated Database of Drug Mechanisms

**DOI:** 10.1038/s41597-023-02534-z

**Published:** 2023-09-16

**Authors:** Adriana Carolina Gonzalez-Cavazos, Anna Tanska, Michael Mayers, Denise Carvalho-Silva, Brindha Sridharan, Patrick A. Rewers, Umasri Sankarlal, Lakshmanan Jagannathan, Andrew I. Su

**Affiliations:** The Scripps Research Institute, Department of Integrative Structural and Computational Biology, 10550 N Torrey Pines Rd, La Jolla, CA 92037 USA

**Keywords:** Data integration, Databases

## Abstract

Computational drug repositioning methods have emerged as an attractive and effective solution to find new candidates for existing therapies, reducing the time and cost of drug development. Repositioning methods based on biomedical knowledge graphs typically offer useful supporting biological evidence. This evidence is based on reasoning chains or subgraphs that connect a drug to a disease prediction. However, there are no databases of drug mechanisms that can be used to train and evaluate such methods. Here, we introduce the Drug Mechanism Database (DrugMechDB), a manually curated database that describes drug mechanisms as paths through a knowledge graph. DrugMechDB integrates a diverse range of authoritative free-text resources to describe 4,583 drug indications with 32,249 relationships, representing 14 major biological scales. DrugMechDB can be employed as a benchmark dataset for assessing computational drug repositioning models or as a valuable resource for training such models.

## Background & Summary

Drug repositioning, the identification of novel uses of existing therapies, has become an increasingly attractive strategy to accelerate drug development^1^. By leveraging available genomics and biomedical domains, computational drug repositioning models have emerged as an unprecedented opportunity to analyze large amounts of data, reducing the time and effort required to identify repositioning candidates.

Computational repositioning models frequently rely on drug-drug and or disease-disease similarity^2,3^. However, the complex and contextual biological associations that underlie the relationship between a drug and a disease often require a more sophisticated explanation. To address this, biomedical knowledge graphs have emerged as a powerful tool capable of capturing biological associations that provide a more comprehensive understanding of the link between a drug and a disease^4^.

Biomedical knowledge graphs consist of nodes representing biological concepts (such as genes, drugs, diseases, and pathways) and edges describing their relationship (such as drugs treating diseases, or diseases being associated with genes)^4^. Repositioning methods based on knowledge graphs leverage the biological associations captured on the network to provide supporting evidence for the model prediction. This is typically achieved by identifying subsets of reasoning chains or subgraphs within the larger network, providing a mechanistic rationale for why a particular drug might be effective against a particular disease, despite the absence of pre-existing evidence to validate the association^5^.

However, one major challenge in determining the plausibility of the supporting evidence provided by biomedical knowledge graphs is the absence of a gold standard, well-defined collection of drug mechanisms. Such a reference point is necessary to evaluate the mechanistic accuracy of predictions made by repositioning models. While validation by domain experts is an alternative approach, it is a laborious and resource-intensive process that demands significant expertise.

Current efforts to construct biomedical networks integrate diverse knowledge bases^5–8^ or extract knowledge from literature using natural language processing techniques^9–11^. However, there are several challenges in creating an accurate and comprehensive knowledge graph that serves as a benchmark for repositioning discoveries. They often lack contextual information, not providing enough information about the relationship between a drug and a disease. Moreover, semantic interoperability is not present in high-quality, where concepts and terminologies within the network are unclear.

To fill this gap, we created Drug Mechanism Database (DrugMechDB), a manually curated database of drug mechanisms expressed as paths through a biomedical knowledge graph. In this work, we present our first complete version of DrugMechDB, comprising 5,666 mechanistic paths that explain 4,583 indications. Each record is derived from free-text descriptions, where each captured concept is normalized to a concept type and mapped to an identifier. We provide a detailed description of the information captured by mechanistic paths, elucidating expressiveness of the database. We assess the quality of association by leveraging an external biomedical knowledge graph. The detailed information contained within DrugMechDB serves as a useful community reference for the development and evaluation of machine learning drug repositioning models. Researches can leverage mechanistic paths of DrugMechDB to enhance the accuracy and effectiveness of their algorithms, leading to more informed decisions.

## Methods

**Fig. 1:**
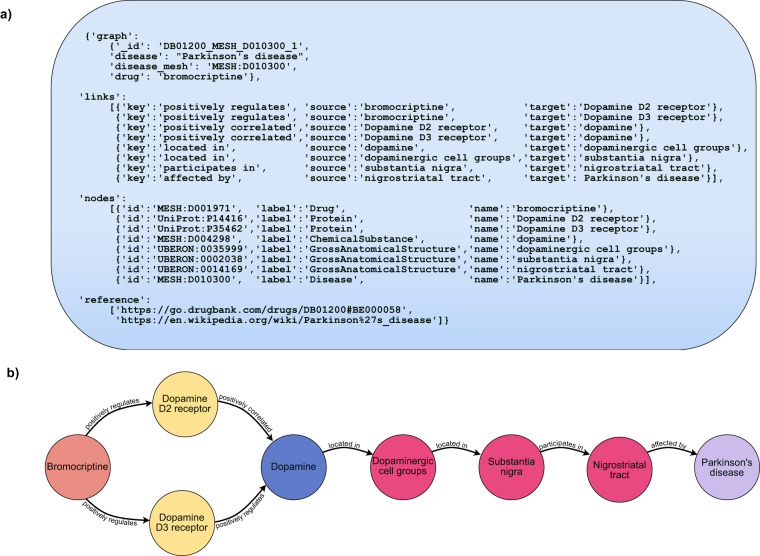
DrugMechDB indication structure. (**a**) Indication JSON formatting. Each record contains several keys that produce a graph that can be programmatically accessed: ‘graph’, ‘links’, ‘nodes’, and ‘reference’. The unique path identifier is included under the graph field (‘_ id’). (**b**) Visualized example of one entry in DrugMechDB: a branching path from Bromocriptine to Parkinson’s disease (‘_ id’: DB01200_MESH_D010300_1).

In DrugMechDB, each curated indication is depicted as a directed graph (Fig. 1). Here, we provide a detailed explanation of the data resources utilized and the curation process undertaken to build DrugMechDB.

### Data sources

DrugMechDB was constructed considering drug-disease indications from the DrugCentral database, using the version downloaded on September 18, 2020^12^. The main source for curation arises from either the Mechanism of Action section from DrugBank^13^, or the Description section within Inxight Drugs^14^. Other resources included review articles, GeneOntology^15,16^, UniProt^17^, Reactome^18^, and well-sources Wikipedia articles^19^, which references were authenticated by curators. Primary literature sources containing experimental results were excluded, ensuring that only highly curated and high-confidence information was included.

### Data model

DrugMechDB provides researchers with a consistent and structured information source on drug mechanisms. To achieve this, we adopted the Biolink Model (version 1.3.0)^20^. The Biolink Model is a standardized hierarchy of biomedical entity classes that serves as a universal framework for biomedical data representation and linkage^21^. It encompasses a wide range of entity types such as genes, proteins, diseases, drugs, and biological processes, and defines the predicates that describe the relationships between these entity types.

The standardization of data in DrugMechDB to the Biolink Model enables the mapping of concepts and relationships to a common vocabulary, thus allowing interoperability between various data sources. Therefore, researchers can easily combine data from DrugMechDB with other biomedical data sources that also employ the same data model, enabling researchers to perform comprehensive analyses and gain new insights into drug mechanisms of action. A list of the DrugMechDB concepts and corresponding relationships is found in Table 1.

**Table 1:** DrugMechDB concept types.

Node types	Abbreviation	Identifier Sources	Unique edge-types	Total edge count
GrossAnatomicalStructure	A	Uber-anatomy ontology (UBERON)^25^	24	534
BiologicalProcess	BP	Gene Ontology (GO)^15,16^	38	8,235
Cell	C	Cell Ontology (CL)^26^	19	186
CellularComponent	CC	Gene Ontology (GO)^15,16^	15	456
Disease	D	Medical Subject Headings (MeSH)	12	147
ChemicalSubstance	CS	Medical Subject Headings (MeSH)	35	2,474
		Chemical Entities of Biological Interest (ChEBI)^27^		
Drug	DX	Medical Subject Headings (MeSH)	38	6,886
		DrugBank^13^		
GeneFamily	G	InterPro^28^, Pfam^29^	21	958
MolecularActivity	M	Gene Ontology (GO)^15,16^	21	474
MacromolecularComplex	MC	Protein Ontology (PR)^30^	1	5
Protein	P	UniProt^17^	33	8,704
PhenotypicFeature	PF	Human Phenotype Ontology (HP)^31^	17	1,499
Pathway	PW	Reactome Pathway (reactome)^18^	20	348
OrganismTaxon	T	NCBITaxon (taxonomy)^32^	5	1,343
			Total	32,249

### Path curation

While free-text descriptions offer a comprehensive narrative of a drug’s mechanism, they can sometimes include information that is not directly relevant to the mechanism of action. Consequently, the process of defining the most suitable relationships that describe a drug’s action can be subjective, resulting in inconsistent annotations. To ensure consistency, accuracy, and clarity among path representations of DrugMechDB records, we established a formal curation guide. Briefly, we ensured to maintain the order of interactions to reflect cause and effect between two concepts, representing the sequence of events or influences. To streamline the paths and eliminate unnecessary complexity, we removed any information that did not significantly contribute to the overall understanding of the drug’s action. Additionally, when multiple related concepts were involved in a sequence of interactions, we summarized them into a single all-encompassing concept, allowing for a more concise and cohesive representation of the drug’s mechanism, reducing redundancy, and improving the clarity of the path.

Lastly, to enhance standardization and minimize inconsistencies in vocabulary conventions, we relied on the Node Normalization service (version 2.1.1)^22^. Each node recorded in DrugMechDB was mapped to the preferred CURIE prefix and label, along with the semantic type defined by the Biolink Model.

## Data Records

The first completed DrugMechDB version (2.0.1)^23^ captures 4,583 curated indications between 1,580 drugs and 744 diseases. DrugMechDB is a knowledge graph with 14 types of nodes and 71 types of directed edges. Currently, it captures 32,588 nodes, and 32,249 edges. We provide a breakdown of the number of edges by concept type in Table 1.

**Fig. 2:**
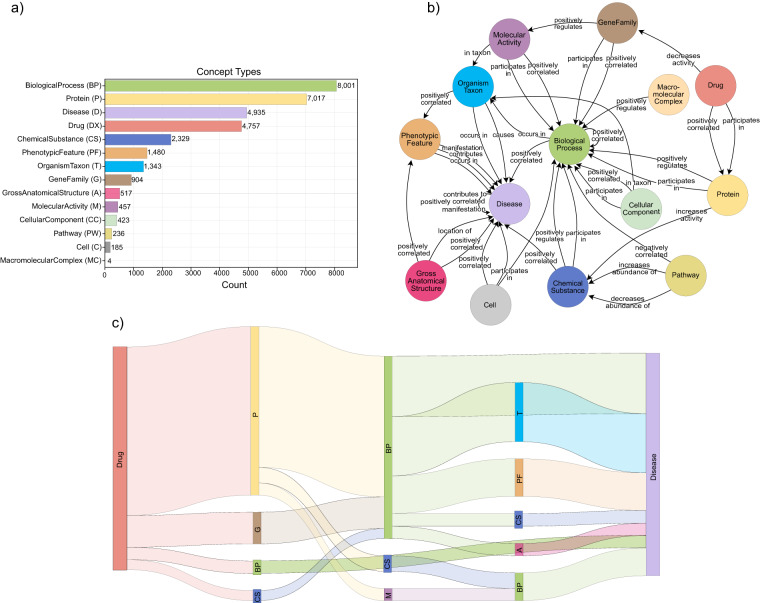
DrugMechDB summary elements. (**a**) Total number of nodes found by concept type. (**b**) Meta-edges of DrugMechDB, displays the top three most representative association types between concept types. (**c**) Sankey diagram depicts the most commonly occurring mechanistic paths, where each rectangle corresponds to a concept type (abbreviated), and the thickness reflects to the number of connections between them.

The number of nodes contained in DrugMechDB by concept type is shown in Fig. 2a, the ‘BiologicalProcess’ concept type appears most frequently as a node on the graph, comprising 24.55 % of the total nodes. Among the total 725 meta-edges, the most common connection occurs between a ‘Protein’ to a ‘BiologicalProcess’ concept type, linked by a ‘positively regulates’ edge type, accounting for 11.29 % of the total meta-edges (Fig. 2b). Each indication is explained through a mechanistic path, a sequence of nodes, and relationships. The current version of DrugMechDB captures a collection of 5,666 curated mechanistic paths. These paths are grouped into 297 distinct types based on the sequence of concept types they encompass (Fig. 2c).

**Fig. 3:**
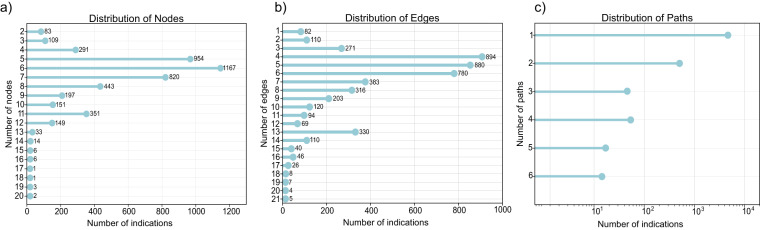
Distribution of DrugMechDB components. Distribution of (**a**) nodes and (**b**) edges across mechanistic paths. (**c**) Distribution of paths that describe curated indications.

The complexity of interactions underlying in drug-disease associations can lead to a wide variation in the number of nodes and edges. Figure 3a,b depict the distribution of the number of nodes and edges captured in DrugMechDB indications, respectively. Some records are relatively simple, with only a few nodes and edges, while others are much more complex, with many interconnected nodes and edges, reflecting the complexity nature of the biological connections. Certain drugs exert their therapeutic effects by engaging in multiple simultaneous interactions. This can entail blocking multiple targets or influencing multiple pathways. In DrugMechDB, such situations are represented by branching paths (Fig. 3c).

All curated records in DrugMechDB are structured in a standardized format, located within the file indication _ paths.json. Each record is represented as a directed graph with the keys: ‘graph’, ‘links’, ‘nodes’, and ‘reference’ (Fig. 1). Indication information, including the drug and disease names and their external identifiers, is captured within ‘graph’ key. Here, we provide a ‘_ id’ value, which is a unique identifier of each record. The relationships and concepts associated to the mechanistic paths of each record are defined within the ‘links’ key. In this key, the ‘source’ and ‘target’ identifiers of the concepts are provided, along with a ‘key’ field that indicates the specific type of relationship between the two nodes. Further information about the concepts in the graph of each record is described within the ‘nodes’ key. Here, each node contains the fields ‘id’, ‘name’, and ‘label’ corresponding to the external identifier, the concept’s name, and the type of concept respectively. Lastly, the ‘reference’ key provides a hyperlink to the data source(s) from which the record was curated.

## Technical Validation

### Systematic validation of DrugMechDB associations

Validating the reliability of a knowledge graph is a crucial step that ensures the correctness of the captured information. In this work, we assessed the accuracy of captured DrugMechDB associations by comparing them to existing data sources. For this, we leverage an external biomedical knowledge graph: Mechanistic Repositioning Network (MechRepoNet)^24^.

Briefly, MechRepoNet is a comprehensive biomedical knowledge graph that was constructed by integrating 18 different data sources and using Biolink Model for standardization. Given that MechRepoNet encompasses a wider network that spans various domains, we employed it as an external benchmark for verifying the plausibility of the associations recorded in DrugMechDB.

Evaluating association types between concept types (ignoring edge predicates), we found that 2,924 (28.71%) of the 10,184 unique associations captured in DrugMechDB are also contained within MechRepoNet. To demonstrate that DrugMechDB associations are broadly consistent with the knowledge captured in MechRepoNet, we conducted a bootstrapping analysis. For each DrugMechDB association type, nonparametric bootstrapping was applied to sample simulated association types (with replacement) to calculate the percentage of matching with MechRepoNet. This procedure was repeated 1,000 times to construct a percentage distribution from which the mean and 99 % CI were calculated. The p-value was calculated as the fraction of the distribution in which the simulated percentage of matching was greater than or equal to the observed percentage. Results in Table 2 show that the average p-value of the ten most frequent association types is less than 0.001, demonstrating that observed overlapping between DrugMechDB and the broader knowledge captured by MechRepoNet is unlikely to occur by chance.

**Table 2:** Validation of the ten most frequent DugMechDB association types.

Association type	DMDB count	MechRepoNet overlap (%)	Mean bootstrapping overlap (99 (%) CI)	P-value
Protein-BiologicalProcess	4,699	40.15	2.21 (1.78–2.66)	<0.001
Drug-Protein	4,475	60.78	4.74 (4.02–5.45)	<0.001
BiologicalProcess-BiologicalProcess	2,889	0.38	0.29 (0.10–0.55)	0.137
Protein-Protein	2,166	2.15	0.09 (0–0.13)	<0.001
BiologicalProcess-Disease	1,897	56.48	39.40 (36.90–41.96)	<0.001
PhenotypicFeature-Disease	1,352	6.41	0.002 (0–0.07)	<0.001
OrganismTaxon-Disease	1,340	30.76	1.37 (0.82–1.94)	<0.001
BiologicalProcess-OrganismTaxon	1,161	11.59	6.08 (4.9–7.40)	<0.001
BiologicalProcess-PhenotypicFeature	1,136	23.59	18.71(16.37–21.12)	<0.001
ChemicalSubstance-BiologicalProcess	972	9.13	3.46 (2.36–4.62)	<0.001

The association type ‘BiologicalProcess’-‘BiologicalProcess’ has the least overlap among the most frequent DrugMechDB association types, highlighting that MechRepoNet does not cover all curated association types of DrugMechDB. To incorporate the missing information in MechRepoNet, we propose using DrugMechDB as a roadmap, helping to prioritize the most significant relationships involved in drug mechanisms and facilitating the integration of biomedical sources.

In summary, DrugMechDB is a comprehensive resource that provides human interpretable explanations when producing computational repositioning predictions, it has the potential to help domain experts to better assess whether a model’s candidate provides enough biological evidence. We believe that DrugMechDB offers several advantages. First, it serves as a useful resource for researchers looking to understand drug pharmacodynamics. Second, it is a valuable training data set that can be incorporated into drug repositioning models that focus on providing supporting plausible reasoning chains. Lastly and as described above, DrugMechDB functions as a roadmap for knowledge graph expansion, helping to prioritize biological associations that most commonly appear in curated drug mechanisms.

## Usage Notes

DrugMechDB provides structured information about drug mechanisms based on a wide range of primary and secondary sources. We believe that DrugMechDB will be a valuable resource for a wide range of computational analyses, including, for example, the identification of drug repositioning candidates. While we are confident in the overall accuracy of the DrugMechDB as a data set for training and/or evaluating machine learning models, we encourage users to critically assess any individual records or assertions used in downstream analyses. Variance could be due to a wide variety of factors, including (but not limited to) differences in data modeling, multiple possible mechanisms described in the literature, and/or errors in structuring knowledge in our curation process.

## Data Availability

The DrugMechDB project website is at https://sulab.github.io/DrugMechDB/. The code to reproduce results, along with curation guidelines, is available in DrugMechDB GitHub repository at https://github.com/SuLab/DrugMechDB/tree/2.0.1. All relevant files are hosted at 10.5281/zenodo.8139357^23^. Additionally, contributions of curated mechanistic paths can be done by pull request to the file submission.yaml at SuLab/DrugMechDB/blob/main/SubmissionGuide.md.
